# The impact of treatment of periodic limb movements in sleep on blood pressure in patients with and without sleep apnea

**DOI:** 10.1038/s41598-022-07659-6

**Published:** 2022-03-07

**Authors:** Daniela Toma, Sebastian Bertram, Diana Racovitan, Maximilian Seidel, Adrian Doevelaar, Felix S. Seibert, Benjamin Rohn, Nina Babel, Dominic Mühlberger, Nikolaus Büchner, Simon Wang, Timm H. Westhoff

**Affiliations:** 1 Medical Department 1, University Hospital Marien Hospital Herne, Ruhr-University Bochum, Hölkeskampring 40, 44625 Herne, Germany; 2 Center for Translational Medicine, University Hospital Marien Hospital Herne, Ruhr-University, Bochum, Germany; 3 Department of Vascular Surgery, University Hospital St. Josef Hospital, Ruhr-University, Bochum, Germany; 4 HELIOS-Klinikum Duisburg-Pneumologie, Schlaf- und Beatmungsmedizin, Duisburg, Germany

**Keywords:** Hypertension, Sleep disorders

## Abstract

Improving sleep quality in patients with obstructive sleep apnea (OSA) by positive airway pressure therapy is associated with a decrease of blood pressure (BP). It remains elusive, whether treatment of sleep disturbances due to restless legs syndrome with symptomatic periodic limb movements in sleep (PLMS) affects BP as well. The present study provides first data on this issue. Retrospective study on patients undergoing polysomnography in a German University Hospital. Inclusion criteria were first diagnosis of restless legs syndrome with PLMS (PLM index ≥ 15/h and PLM arousal index ≥ 5/h) with subsequent initiation of levodopa/benserazide or dopamine agonists. Exclusion criterion was an initiation or change of preexisting positive airway pressure therapy between baseline and follow-up. BP and Epworth sleepiness scale were assessed at two consecutive polysomnographies. After screening of 953 PLMS data sets, 114 patients (mean age 62.1 ± 12.1 years) were included. 100 patients (87.7%) were started on levodopa/benserazide, 14 patients (12.2%) on dopamine agonists. Treatment was associated with significant reductions of PLM index (81.2 ± 65.0 vs. 39.8 ± 51.2, p < 0.001) and ESS (6 [interquartile range, IQR, 3–10.5] vs. 5 [IQR 3–10], p = 0.013). Systolic BP decreased from 132.9 ± 17.1 to 128.0 ± 15.8 mmHg (p = 0.006), whereas there was no significant change of diastolic BP (76.7 ± 10.9 vs. 75.1 ± 9.2 mmHg, p = 0.15) and heart rate (71.5 ± 11.9 vs. 71.3 ± 12.7, p = 0.84). The number of antihypertensive drugs remained unchanged with a median of 2 (IQR 1–3, p = 0.27). Dopaminergic treatment of PLMS is associated with an improvement of sleep quality and a decrease of systolic BP comparable to treatment OSA.

## Introduction

Obstructive sleep apnea and periodic limb movements in sleep (PLMS) are associated with increased cardiovascular risk including hypertension^1–3^. Treatment of obstructive sleep apnea by continuous positive airway pressure (CPAP) is associated with a decrease in blood pressure. Metaanalyses show a mean reduction of 3 mmHg in systolic and 2 mmHg in diastolic blood pressure (BP)^4^. This effect occurs even in resistant hypertension and increases with baseline blood pressure, young age, and the severity of oxygen desaturations^5,6^. The effects of CPAP exceed the blood pressure lowering effects of nocturnal oxygen supplementation^7^. Whereas the effects of treatment of obstructive sleep apnea on BP are thus well defined, the effects of treatment of restless legs syndrome with PLMS remain elusive.

There is evidence linking PLMS to hypertension, but the underlying mechanisms are unclear. Since the nocturnal BP of patients with PLMS is higher than those with insomnia, it may be suggested that PLMS affects autonomic regulation beyond a mere disturbance of sleep^8^. This assumption is supported by the finding that limb movements in sleep are associated with sudden increases of heart rate and BP even in otherwise healthy subjects^1^. First data on the vasoregulatory effects of dopaminergic treatment are controversial. Whereas pramipexole did not affect autonomic balance during wakefulness in a small study of 20 patients with periodic leg movements, rotigotine at optimal doses (1–3 mg/24 h) showed some effect on nocturnal systolic BP^9,10^. With regard to these conflicting data, it remains unclear, whether the proposed rotigotine effect is substance-specific and whether dopaminergic treatment is able to affect daytime BP as well.

The present retrospective analysis for the first time examines the effect of dopaminergic treatment of PLMS on office BP.

## Methods

### Patients and protocol

We performed a retrospective study of polysomnographic recordings and clinical data of patients undergoing in-laboratory polysomnography in a German University Hospital. We made use of an electronic data extraction approach to identify subjects suffering from a restless legs syndrome with PLMS that had been admitted to our institution from 2010 to 2020. Inclusion criteria were clinical features of restless legs syndrome with first diagnosis of PLMS (periodic leg movement index ≥ 15/h and periodic leg movement arousal index ≥ 5/h) and subsequent initiation of therapy with dopamine agonists or levodopa/benserazide. Exclusion criterion was an initiation or change of preexisting positive airway pressure therapy between baseline and follow-up polysomnography. Clinical data were taken at baseline polysomnography prior to initiation of therapy for PLMS. Follow-up polysomnography was conducted after a median of 8 months after initiation of therapy. The daytime sleepiness was assessed using the Epworth Sleepiness Scale (ESS) questionnaire. BP and ESS were assessed at baseline and follow-up. Polysomnographic monitoring comprised simultaneous noninvasive recordings of electroencephalogram, electrooculogram, submental and bilateral leg electromyogram, nasal or oral airflow, oxygen saturation (pulse oximetry), snoring microphone, chest and abdominal respiratory movement, body position and electrocardiogram. Polysomnography results were scored according to standard criteria according to American Academy of Sleep Medicine guidelines by an experienced observer^11,12^. Results were interpreted by physicians with long-lasting experience in sleep medicine. Hypertension was defined as either systolic office blood pressure > 140 mmHg, diastolic blood pressure of > 90 mmHg, or use of antihypertensive medication. The study was approved by the ethical committee of Ruhr-University Bochum (Reference number 18-6725-BR).

### Assessment and treatment of restless legs with periodic limb movements in sleep

PLMS was assessed and defined according to the American Sleep Disorders Association criteria (10) as bursts of muscle activity on the anterior tibialis electromyogram of 0.5–10 s duration, > 25% of the amplitude of a voluntary movement, and as a part of a series of ≥ 4 movements separated by 5–90 s^13^. According to the official World Association of Sleep Medicine (WASM) standards for recording and scoring periodic leg movements in sleep PLM is associated with respiratory events and should not be scored when an overlap respiratory event and PLM occurred or the interval between PLM and the respiratory event is less than 0.5 s, regardless which event is first^13–15^. The PLM frequency was quantified by a periodic movement index, calculated as the number of movements per hour of total sleep time, with ≥ 5 movements per hour considered abnormal^13,14^. The movement-related arousal index was calculated as the number of PLMS related arousals per hour of total sleep time. According to our center’s standard we start treatment with levodopa/benserazide (start dosis 100/25 mg) in patients > 60 years and dopamine agonists in you younger patients (0.18 mg pramipexole or 1 mg ropinirole per day). Thereafter, patients were uptitrated to their optimal dose by their general practitioner or neurologist.

### Blood pressure measurement

Office blood pressure was assessed auscultatorily according to Riva-Rocci at admission by trained observers in a seated position in a quiet room of the laboratory after a resting time of ≥ 5 min. Measurements were performed at daytime during a physical examination prior to the polysomnography. The cuff size was chosen in dependence of the upper arm circumference using a standard bladder cuff 13 × 24 cm for a circumference of 24–32 cm, 15 × 30 cm for a circumference of 33–41 cm, and 12 × 22 cm for a circumference of < 22 cm. The cuff was deflated by a maximum of 3 mmHg per second. Two measurements were obtained and the mean of these measurements was used for further analysis.

### Sample size estimation

With regard to studies on the effect of CPAP on blood pressure in subjects with obstructive sleep apnea, we anticipated an effect of 3 mmHg on systolic blood pressure with a standard deviation of 10 mmHg for the sample size calculation. Based on a paired two-tailed *t* test, a study size of 90 is necessary to achieve a power of 80% and a two-sided level of significance of 5%, for detecting this difference in blood pressure. We aimed to exceed this number by > 20% for drop-outs due to incomplete study data.

### Statistics

Data were checked for normality of distribution by the Shapiro–Wilk Test. Since both data on blood pressure and periodic limb movements turned out to be normally distributed, measures at baseline were compared to follow-up by a paired two-tailed *t* test. Age, ESS score, and number of antihypertensive drugs are presented as median and interquartile range (IQR). Baseline and follow-up ordinal data were analyzed by the Wilcoxon signed rank test. Dichotomous parameters were compared by Chi-squared test. Association of baseline systolic BP, PLMI, ESS score and change of systolic BP from baseline to follow-up was analyzed by univariate linear regression analysis. P < 0.05 was regarded statistically significant. All statistical analysis was done using SPSS Statistics 25 (SPSS Inc, Chicago, Illinois, USA).

### Ethics approval and consent to participate

This study was conducted in accordance with the declaration of Helsinki and was approved by the ethics committee of Ruhr-University Bochum (Reference number 18-6725-BR). The need for patient approval and informed consent was waived by ethics committee of Ruhr-University Bochum due to the retrospective nature of the study.

## Results

After screening of 953 data sets, 114 fulfilled all in- and exclusion criteria and were included in the analysis. The study population had a median age of 63 (IQR 52–72.5). 47 patients (41.2%) of the study population were female. 90 patients (78.9%) had hypertension. The median number of antihypertensive drugs in the overall study population was 2 (IQR 1–3) at baseline and follow-up. The most prevalent further cardiovascular comorbidities were diabetes (n = 27, 23.8%), coronary heart disease (n = 23, 20.0%) and hyperlipidemia (n = 60, 52.6%). Epidemiological and clinical baseline parameters are presented in Table 1.

**Table 1:** Epidemiological and clinical baseline parameters of the study population.

	Study population (n = 114)
Age (years)	63 (IQR 52 – 72.5)
**Gender**	
Female	47 (41.2%)
Male	67 (58.8%)
**Cardiovascular disease**	
Hypertension	90 (78.9%)
Number of antihypertensive drugs (median and IQR)	2 (IQR 1–3)
Baseline systolic office blood pressure (mmHg)	132.9 ± 17.1
Baseline diastolic office blood pressure (mmHg)	75.9 ± 12.5
Diabetes	27 (23.8%)
Coronary heart disease	23 (20%)
Hyperlipidemia	60 (52.6%)
**Sleep disorders**	
Obstructive sleep apnea	98 (85.9%)
Established CPAP therapy	74 (64.9%)
Apnea hypopnea index (1/h)	4.6 ± 11.8
Periodic limb movement index (1/h)	81.2 ± 65.0
Epworth sleepiness scale score	6 (IQR 3–10.5)

40 (35.1%) of the study population underwent their first polysomnographic examination. The diagnosis of obstructive sleep apnea was preestablished in 74 (64.9%) and 24 (21.1%) were found to have obstructive sleep apnea at baseline investigation. 74 (64.9%) underwent polysomnography with established CPAP therapy. Baseline median ESS index was 6 (IQR 3–10.5). All the patients were diagnosed to have PLMS and treatment was initiated. At follow-up, 100 patients (87.7%) were administered levodopa/benserazide and 14 patients (12.2%) dopamine agonists (pramipexole or ropinirole). Mean levodopa/benserazide dosage was 159.0 ± 67.8 mg ranging from 62.5 to 375 mg. In 12 (10.5%) patients, pramipexole was initiated in a dosage of 0.08 to 0.52 mg (mean 0.3 ± 0.1 mg). Ropinirole was used in two (1.7%) patients in a dosage of 1 to 4 mg (mean 2.5 ± 1.5 mg).

**Figure 1:**
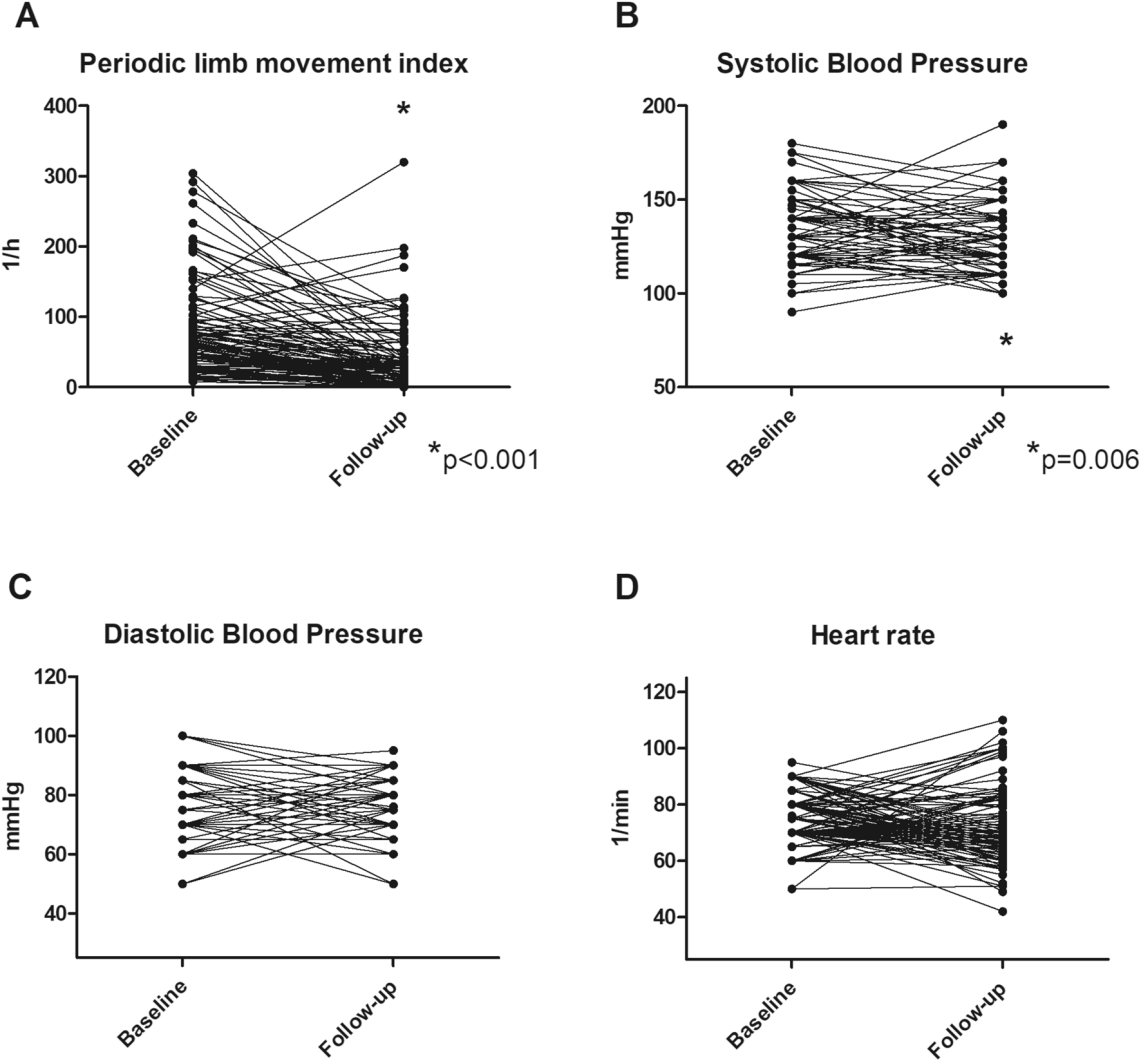
Individual course of (**A**) periodic limb movement index (PLM index), (**B**) systolic office blood pressure (SBP), (**C**) diastolic office blood pressure (DBP), and (**D**) heart rate from baseline to follow-up.

Table 2 presents data on PLM index (PLMI), systolic and diastolic office blood pressure, heart rate, apnea hypopnea index, and ESS index at baseline and follow-up. The individual courses of blood pressure, heart rate, and PLMI are illustrated by Fig. 1. At baseline, PLMI was 81.2 ± 65.0/h. Dopaminergic treatment of PLMS was initiated in all the patients.

**Table 2:** Sleep disorder parameters and office blood pressure at baseline and follow-up. Numeric data were compared by two-tailed *t* tests, ordinal data (ESS) by the Wilxocon signed rank test. P < 0.05 was regarded significant. *PLMI* periodic limb movement index, *ESS* Epworth Sleepiness Scale, *AHI* apnea hypopnea index, *BP* blood pressure.

	Baseline	Follow-up	P
PLMI (1/h)	81.2 ± 65.0	39.8 ± 51.2	**< 0.001**
ESS index	6 (IQR 3–10.5)	5 (IQR 3–10)	**0.01**
AHI (1/h)	4.6 ± 11.8	4.3 ± 10.9	**0.42**
Systolic office BP (mmHg)	132.9 ± 17.1	128.0 ± 15.8	**0.006**
Diastolic office BP (mmHg)	76.7 ± 10.9	75.1 ± 9.2	**0.15**
Heart rate (1/min)	71.5 ± 11.9	71.3 ± 12.7	**0.84**
**Dopaminergic therapy**			
Levodopa/benserazide (mg)	–	159 ± 67.8	
Pramipexole (mg)	–	0.3 ± 0.1	
Rotigotine (mg)	–	2.5 ± 1.5

**Figure 2:**
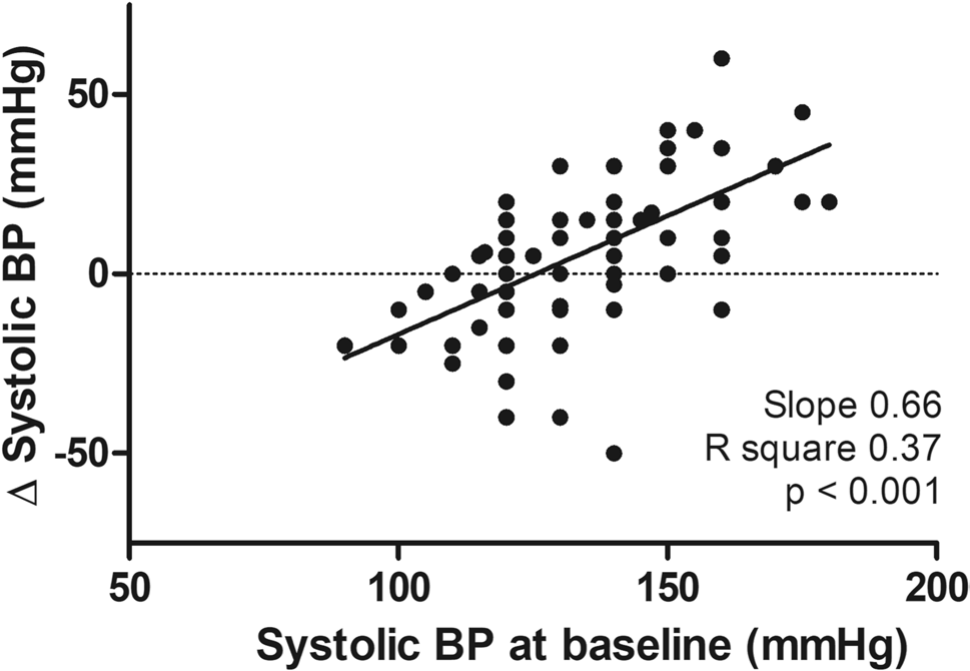
Change of systolic blood pressure (BP) from baseline to follow-up in dependence of baseline systolic blood pressure (linear regression analysis).

AHI did not significantly differ from baseline to follow-up (4.6 ± 11.8/h vs. 4.3 ± 10.9/h, p = 0.42). On the contrary, PLMI decreased significantly from 81.2 ± 65.0/h to 39.8 ± 51.2/h, p < 0.001. ESS was significantly reduced from 6 (IQR 3–10.5) to 5 (IQR 3–10) p = 0.013. Systolic office BP was significantly diminished from 132.9 ± 17.1 mmHg to 128.0 ± 15.8 mmHg, p = 0.006. The change of systolic BP from baseline to follow-up significantly depended on baseline systolic BP (Fig. 2). The higher baseline BP, the more BP reduction by dopaminergic therapy. Linear regression analysis revealed a regression coefficient b of 0.66, an R squared of 0.37 and p < 0.0001. Univariate regression analysis demonstrated no significant impact of baseline PLMI (b = 0.047, p = 0.08) or ESS index (b = − 0.017, p = 0.961) on change of systolic BP. Finally, neither diastolic BP (76.7 ± 10.9 mmHg vs. 75.1 ± 9.2 mmHg, p = 0.48), nor heart rate (71.5 ± 11.9/min vs. 71.3 ± 12.7/min; p = 0.84) did significantly differ from baseline to follow-up.

## Discussion

Treatment of obstructive sleep apnea by continuous positive airway pressure (CPAP) contributes to BP control in hypertensive patients. The present study shows for the first time that treatment of restless legs with PLMS is associated with a significant decrease of systolic BP as well. The mean reduction of systolic BP was 5 mmHg, which numerically exceeds the average BP lowering effects of positive airway pressure therapy in sleep apnea of about 3 mmHg^4^. The extent of BP reduction crucially depends on baseline BP. The higher baseline systolic BP, the higher the decrease of BP. This phenomenon exists in the same way for other pharmacological and non-pharmacological interventions to reduce BP in hypertension. Thus, antihypertensive drugs are more effective in subjects with higher baseline BP levels. In analogy, the exercise-induced effects on BP strongly depend on baseline systolic BP^16^. Analogously, continuous positive airway pressure application in sleep apnea lowers BP more effectively in hypertensive than in normotensive subjects^4^. Noteworthy, baseline systolic BP was only 133 mmHg in the present study population. Thus, hypertension was frequent (79%) but well managed by pharmacological treatment prior to administration of dopaminergic therapy. Therefore, the present BP lowering effects of treatment of PLMS are indeed comparable or even exceed the effects of positive airway pressure therapy in sleep apnea.

In contrast to systolic BP, treatment of PLMS did not reveal a significant effect on diastolic BP. Whereas somewhat surprising at first sight, this finding fits to general experiences with antihypertensive interventions as well. Thus, the antihypertensive effects on diastolic BP are inferior to systolic BP in other pharmacological and non-pharmacological interventions as well including positive airway therapy in sleep apnea^4,16–19^.

The reduction of BP did not significantly depend on baseline PLMI and ESS. This finding contrasts data on application of positive airway pressure in obstructive sleep apnea. Here, the effects on BP are more pronounced in subjects with higher AHI and sleepiness^20,21^. The reasons for this diverging finding remain elusive. On the one hand, the study size may have been too small to detect a significant finding. Noteworthy, there is indeed a non-significant trend to higher effects on BP in subjects with higher PLMI. On the other hand, the BP reduction may partially be mediated by BP-lowering side effects on levodopa/benserazide and dopamine agonists independent of an improvement of sleep quality.

86% of the patients in this study had coincident obstructive sleep apnea. As requested by the exclusion criteria, however, none of these subjects had an initiation, nor a change of preexisting positive airway pressure therapy from baseline to follow-up. Thus, it can be ruled out that CPAP therapy was responsible for the observed changes of BP from baseline to follow-up. Accordingly, AHI did not significantly differ between baseline and follow-up. Moreover, the number of antihypertensive drugs did not differ between baseline and follow-up.

PLMS is associated with increased sympathetic activity. A previous study showed increased sympathetic activity in PLMS^22^. Specifically, a change in heart rate variability and cortical activation was shown to reflect sympathetic activity^22^. Thus, a reduction of sympathetic activity might contribute to the decrease of BP. Whether the reduction in the number of leg movements or CPAP therapy contributed to the reduction in blood pressure cannot be conclusively assessed in this study.

Treatment of PLMS reduced systolic BP by a mean of 5 mmHg in the present population. This reduction has to be regarded as clinically relevant. Even a 1 to 2 mmHg reduction in BP is associated with a reduction in major cardiovascular events, stroke, and especially heart failure^19^. In the Syst-Eur trial, placebo and treatment group differed in systolic BP by 10 mmHg and the incidence of stroke was reduced by 42%^23^. Thus, a 5 mmHg reduction may be associated with a relative risk reduction of approximately 20%.

The study is limited by its BP measurement technique, its study size and its retrospective design. We made use of office BP as primary endpoint. Ambulatory blood pressure monitoring (ABPM) would have ruled out a white coat effect, would have had a higher prognostic value and would have provided insight into nighttime BP^24^. Noteworthy, however, office BP is closely correlated to ABPM and constitutes the diagnostic standard in polysomnography laboratories^25^.

The small study results from a very strict definition of inclusion and exclusion criteria. These strict criteria were a crucial prerequisite to exclude effects of changes in pharmacological antihypertensive therapy or treatment of sleep apnea on BP. Regarding the study design, a prospective randomized study design would have necessitated to withhold therapy of PLMS in 50% of the symptomatic study population, which would be ethically disputable. For the same reason, it was not able to provide a control group in this retrospective analysis.

In conclusion, the present study shows for the first time that dopaminergic treatment of restless legs with PLMS is associated with a clinically relevant decrease of systolic BP. The reduction of 5 mmHg is comparable or even exceeds the effects of treatment of sleep apnea and is larger in subjects with higher baseline BP. Future studies should investigate the effects of PLMS treatment on 24 h ambulatory BP.

## References

[1] Pennestri MH (2013). Blood pressure changes associated with periodic leg movements during sleep in healthy subjects. Sleep Med..

[2] Winkelman JW, Shahar E, Sharief I, Gottlieb DJ (2008). Association of restless legs syndrome and cardiovascular disease in the Sleep Heart Health Study. Neurology.

[3] Winkelman JW, Finn L, Young T (2006). Prevalence and correlates of restless legs syndrome symptoms in the Wisconsin Sleep Cohort. Sleep Med..

[4] Fava C (2014). Effect of CPAP on blood pressure in patients with OSA/hypopnea a systematic review and meta-analysis. Chest.

[5] Martinez-Garcia MA (2013). Effect of CPAP on blood pressure in patients with obstructive sleep apnea and resistant hypertension: The HIPARCO randomized clinical trial. JAMA.

[6] Pengo MF (2020). Obstructive sleep apnoea treatment and blood pressure: Which phenotypes predict a response? A systematic review and meta-analysis. Eur. Respir. J..

[7] Gottlieb DJ (2014). CPAP versus oxygen in obstructive sleep apnea. N. Engl. J. Med..

[8] Sieminski M, Chwojnicki K, Partinen M (2017). Higher nocturnal systolic blood pressure in patients with restless legs syndrome compared with patients with insomnia. Sleep Med..

[9] Bauer A (2016). Rotigotine's effect on PLM-associated blood pressure elevations in restless legs syndrome: An RCT. Neurology.

[10] Rocchi C (2015). Chronic dopaminergic treatment in restless legs syndrome: Does it affect the autonomic nervous system?. Sleep Med..

[11] Hori T (2001). Proposed supplements and amendments to 'A Manual of Standardized Terminology, Techniques and Scoring System for Sleep Stages of Human Subjects', the Rechtschaffen & Kales (1968) standard. Psychiatry Clin. Neurosci..

[12] Berry RB (2012). Rules for scoring respiratory events in sleep: Update of the 2007 AASM Manual for the Scoring of Sleep and Associated Events. Deliberations of the Sleep Apnea Definitions Task Force of the American Academy of Sleep Medicine. J. Clin. Sleep Med..

[13] Recording and scoring leg movements. The Atlas Task Force. *Sleep* **16**, 748–759 (1993). 8165390

[14] Zucconi M (2006). The official World Association of Sleep Medicine (WASM) standards for recording and scoring periodic leg movements in sleep (PLMS) and wakefulness (PLMW) developed in collaboration with a task force from the International Restless Legs Syndrome Study Group (IRLSSG). Sleep Med..

[15] Allen RP (2003). Restless legs syndrome: diagnostic criteria, special considerations, and epidemiology. A report from the restless legs syndrome diagnosis and epidemiology workshop at the National Institutes of Health. Sleep Med..

[16] Westhoff TH (2007). Too old to benefit from sports? The cardiovascular effects of exercise training in elderly subjects treated for isolated systolic hypertension. Kidney Blood Press. Res..

[17] Dimeo F (2012). Aerobic exercise reduces blood pressure in resistant hypertension. Hypertension.

[18] Cornelissen VA, Smart NA (2013). Exercise training for blood pressure: A systematic review and meta-analysis. J. Am. Heart Assoc..

[19] Turnbull F, Blood Pressure Lowering Treatment Trialists Consortium (2003). Effects of different blood-pressure-lowering regimens on major cardiovascular events: Results of prospectively-designed overviews of randomised trials. Lancet.

[20] Parati G, Lombardi C (2010). Control of hypertension in nonsleepy patients with obstructive sleep apnea. Am. J. Respir. Crit. Care Med..

[21] Barbe F (2012). Effect of continuous positive airway pressure on the incidence of hypertension and cardiovascular events in nonsleepy patients with obstructive sleep apnea: A randomized controlled trial. JAMA.

[22] Guggisberg AG, Hess CW, Mathis J (2007). The significance of the sympathetic nervous system in the pathophysiology of periodic leg movements in sleep. Sleep.

[23] Staessen JA (1997). Randomised double-blind comparison of placebo and active treatment for older patients with isolated systolic hypertension. The Systolic Hypertension in Europe (Syst-Eur) Trial Investigators. Lancet.

[24] Dolan E (2005). Superiority of ambulatory over clinic blood pressure measurement in predicting mortality: The Dublin outcome study. Hypertension.

[25] Ram CVS (2021). Correlation between ambulatory blood pressure monitoring and office blood pressure measurement in patients with hypertension: A community study. Am. J. Med. Sci..

